# RNA-Mediated Inhibition Mechanism of Liquid–Liquid Phase Separation and Subsequent Aggregation Revealed by Raman Microscopy

**DOI:** 10.1021/jacsau.5c01234

**Published:** 2025-11-03

**Authors:** Taisei Ogura, Uchu Matsuura, Masato Machida, Kaichi Nagai, Shinji Kajimoto, Shinya Tahara, Takakazu Nakabayashi

**Affiliations:** Graduate School of Pharmaceutical Sciences, 13101 Tohoku University, Aoba-Ku, Sendai 980-8578, Japan

**Keywords:** Liquid−liquid phase separation, Protein aggregation, Neurodegenerative diseases, RNA, Raman microscopy

## Abstract

Liquid–liquid phase separation (LLPS) is a phenomenon where homogeneous solutions of biomacromolecules separate into two liquid phases and generate liquid droplets enriched in specific biomolecules. LLPS of neurodegeneration-related proteins, including fused in sarcoma (FUS), promotes their aggregation, causing fatal diseases such as amyotrophic lateral sclerosis (ALS). Recent studies showed that RNAs regulate LLPS of these proteins and inhibit their aggregation, which may play an important role in preventing the disease onset; however, the underlying molecular mechanisms remain elusive. It is also unknown whether endogenous RNAs regulate LLPS and subsequent aggregation in cells. In this study, we investigated features of RNAs that enable their entrance into FUS droplets and inhibition of FUS aggregation via droplets and clarified the underlying mechanisms using Raman microscopy. We found that RNA length is one of the primary factors governing both the aggregation-inhibition effect and the localization of RNAs in the droplets in buffer solutions. Short (<50-nt) RNAs were concentrated inside the droplets and inhibited the aggregation. Our quantification method using Raman microscopy revealed that the short RNAs are enriched in FUS droplets by binding to FUS proteins through electrostatic interactions. On the other hand, long (>1000-nt) RNAs were not concentrated and dissolved the droplets. Raman imaging of living cells revealed that intracellular FUS droplets are enriched with endogenous RNAs at levels comparable to in vitro droplets and exhibit high fluidity, confirming that endogenous RNAs play a crucial role in suppressing droplet-to-aggregate transition of FUS in cells. These findings indicate that short RNAs stabilize FUS droplets through heterotypic RNA–FUS interactions that compete with homotypic FUS–FUS direct contacts responsible for aggregation, whereas binding of long RNAs enhances FUS solubility and promotes droplet dissolution. Our study highlights the protective role of RNAs against pathogenic aggregation of neurodegeneration-related proteins via droplets.

## Graphical abstract

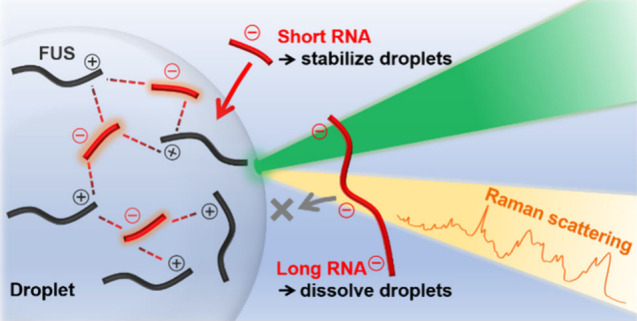

## Introduction

Membraneless organelles and various granular structures within cells are now recognized as liquid droplets formed through liquid–liquid phase separation (LLPS).^1,2^ LLPS drives the demixing of a homogeneous solution of biomacromolecules into two or more distinct liquid phases, resulting in droplets enriched with specific biomolecules. Proteins that undergo LLPS typically contain a certain length of an intrinsically disordered region (IDR), and weak intramolecular interactions, such as cation-π and hydrophobic interactions, between IDRs are considered as the driving force of LLPS.^3,4^ Droplets assemble or exclude specific biomolecules, thereby enabling spatiotemporal regulation of intracellular reactions;^5^ however, LLPS can also contribute to cellular dysfunction.^6,7^ For example, the formation of protein aggregates implicated in neurodegenerative diseases, such as amyotrophic lateral sclerosis (ALS) and Parkinson’s disease, is proposed to be markedly promoted inside the droplets.^8–15^ In fact, various neurodegeneration-related proteins have been reported to form liquid droplets via LLPS, which can subsequently transition into irreversible aggregates.^11,16–21^ Liquid droplets formed can thus be regarded as precursors of the pathological aggregates.

It has been proposed that aggregates of fused in sarcoma (FUS), which are implicated in ALS pathogenesis, are formed via liquid droplets. FUS is one of the RNA-binding proteins (RBPs) with multiple functions^22–25^ that effectively induces LLPS. Cell-derived and yeast RNAs promote LLPS of FUS below a specific RNA concentration, whereas excessive RNA concentrations lead to the dissolution of the droplets.^26,27^ FUS is homogeneously distributed in the nucleus of HeLa cells; however, degradation of nuclear RNA causes the formation of FUS droplets, suggesting that the nuclear RNA acts to inhibit LLPS of FUS,^26^ which is considered to be one of the key mechanisms that prevents its aggregation and the subsequent onset of ALS. RNA-mediated promotion or inhibition of LLPS has also been reported for other RBPs such as TDP-43, hnRNPA1, tau, and the nucleocapsid protein of SARS-CoV-2.^28–31^

Insights into the molecular mechanisms by which RNA regulates LLPS remain limited. Several quantitative studies have shown that the balance of electric charge in droplets governs the promotion and inhibition of LLPS.^32,33^ Notably, the liquid droplets formed by DEAD-box helicase (DDX4)^34^ selectively incorporate or exclude RNAs depending on their hybridization state,^35^ underscoring the importance of RNA structures in mediating protein-RNA interactions within droplets. Thus, evaluating how RNA structural diversity influences RNA localization within droplets and subsequent droplet behaviors is essential for elucidating the mechanisms of RNA-mediated LLPS regulation. Moreover, understanding whether endogenous RNAs are indeed present in droplets in living cells is crucial for elucidating the physiological roles of RNAs in the droplets; however, such an investigation remains limited due to the lack of suitable methods.

Raman microscopy enables label-free, nondestructive analysis^36^ of liquid droplets as a single droplet state. This technique provides structural information on the constituents inside droplets, including aggregation-related changes in protein structures and hydrogen-bonding networks of water molecules.^37–44^ We demonstrated that Raman microscopy can determine concentrations of various biomolecules within individual droplets both in buffers and cells.^19,41–45^ Raman microscopy is thus a powerful method for investigating whether RNA is present in a droplet, its quantity, and its role in droplet properties without the need for labeling or destruction.^45^

In this study, we investigated what specific features of RNAs enable their entrance into FUS droplets and the suppression of aggregation using Raman microscopy. Irrespective of sequence, short-chain RNAs were found to be enriched within the droplets and inhibited aggregation, whereas long-chain RNAs were not enriched and instead dissolved the droplets. Furthermore, we showed that FUS droplets in cells contain substantial amounts of endogenous RNA, with the RNA concentration inside intracellular droplets being consistent with that in buffer solutions. These findings indicate that RNA plays a crucial role in suppressing the aggregation of FUS droplets.

## Results and Discussion

### RNAs Regulate FUS LLPS and Aggregation in an RNA-Length-Dependent Manner

We investigated LLPS and the subsequent aggregation of FUS in the presence of RNAs with different lengths and sequences. The protocols on sample preparation and all the following measurements are described in the Supporting Information. We prepared five RNAs. pncRNA31, cRNA, and polyU are short-chain 31-nucleotide (nt) RNAs and have a sequence different from each other (Figure S1). pncRNA31 has a hairpin structure and selectively binds to FUS.^46^ cRNA has a sequence obtained by permutating the sequence of pncRNA31. PolyU is a disordered RNA composed solely of uracil. pncRNA50 is a 50-nt partial sequence of pncRNA, which has an additional 19 nts at the 3′ end of pncRNA31 (Figure S1). nRNA is a mixture of RNAs whose typical length exceeds 1000 nts, extracted from the nucleus of HeLa cells.

We acquired bright-field images of FUS solutions in the presence and absence of RNAs at incubation times of 0 and 1 h (Figure 1). In the absence of RNAs, droplets with diameters of ∼10 μm were observed at 0 h (Figure 1A). After 1 h incubation, FUS showed aggregate-like structures. 1,6-Hexanediol (16HD) is commonly used to confirm the liquid property of droplets, and droplets formed by hydrophobic interactions are dissolved by 16HD, while solid aggregates remain undissolved. A 10 w/w% solution of 16HD did not completely dissolve the FUS assembly (Figure 1A), confirming the formation of solid-like FUS aggregates after 1 h.

**Figure 1:**
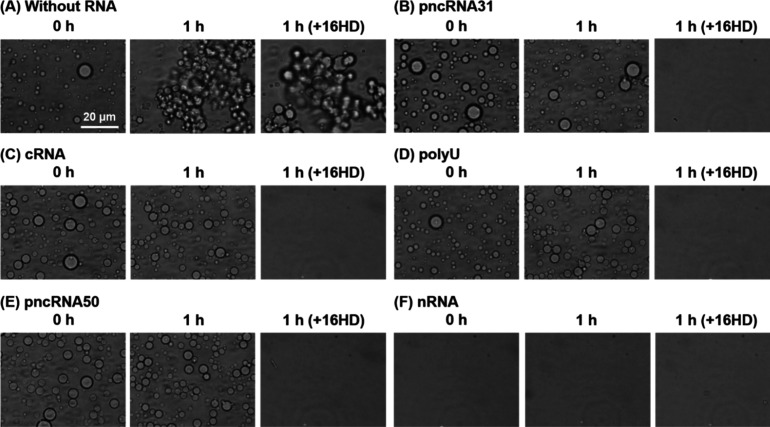
Bright-field images of FUS in buffer solutions as a function of incubation time (A) without RNAs and with (B) pncRNA31, (C) cRNA, (D) polyU, (E) pncRNA50, and (F) nRNA. The images after 1 h incubation and subsequent 1,6-hexanediol (16HD) treatment are also shown. Without RNAs, droplets were observed at 0 h, and after 1 h, aggregates that cannot be dissolved by 16HD were generated. With the RNAs, droplets were retained even after 1 h or no droplets were formed.

In the presence of 1000 ng/μL of pncRNA31, cRNA, polyU, and pncRNA50, the FUS droplets maintained their spherical shapes even after 1 h incubation (Figure 1B-E), and these droplets were almost completely dissolved by 16HD. This result indicates that these RNAs inhibit the transition of FUS from a liquid-like droplet to an aggregate state. This conclusion is supported by the turbidity assay, which showed that FUS solutions incubated for 1 h and treated with 16HD exhibited lower turbidity in the presence of these RNAs compared with that without RNAs (Figure S2). In contrast, 1000 ng/μL nRNA readily dissolved the droplets at 0 h, and no aggregation was observed after 1 h incubation (Figure 1F), which is qualitatively consistent with a previous study reporting that the addition of cell-derived RNAs leads to the disappearance of FUS droplets.^26^ The droplet dissolution with the addition of nRNA was confirmed by the marked decrease in the turbidity of the FUS solution (Figure S2). In summary, all of the prepared RNAs suppressed the aggregate formation from the droplets; however, 50-nt or shorter RNAs stabilized the spherical droplets, while those longer than 1000 nts caused the droplet dissolution.

### RNAs Are Concentrated in the Liquid Droplets in an RNA-Length-Dependent Manner

To investigate whether RNAs are incorporated into FUS droplets, we measured Raman spectra inside the droplets using a Raman microscope (Figure 2). In the absence of RNAs, the Raman spectrum of the droplets exhibited strong bands due to FUS, such as the bands of tyrosine residues at 640 and 1616 cm^–1^, the amide I band at 1669 cm^–1,^ and the C–H stretching band around 2940 cm^–1^ (Figures 2A and S3A, the bands indicated with F arise from FUS). The Raman spectrum acquired outside the droplets is absent from bands attributable to FUS (Figure S4), indicating that FUS is highly concentrated within the droplets. We quantified the FUS concentration within the droplets using a previously established method.^41^ We used the calibration line constructed from Raman measurements of homogeneous FUS solutions at various concentrations (Figure S5A). Using this approach, the FUS concentration inside the droplets was evaluated to be 7.5–8.5 mM, which is comparable to the concentration evaluated by a previous NMR study in an agarose hydrogel (6.8 mM).^47^

**Figure 2:**
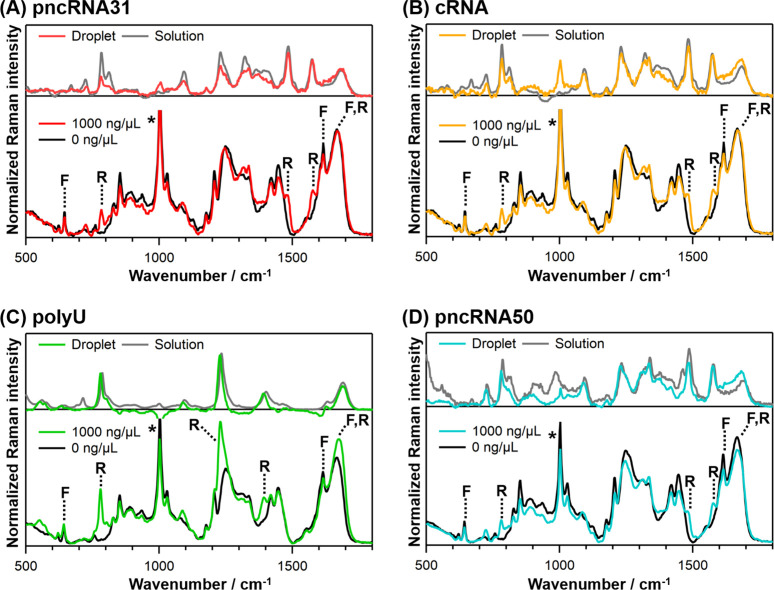
Raman spectra inside FUS droplets in the presence and absence of 1000 ng/μL (A) pncRNA31, (B) cRNA, (C) polyU, and (D) pncRNA50. All the spectra were normalized by the Raman intensity of water outside droplets at around 3400 cm^–1^. The Raman bands indicated with F and R arise from FUS and the RNAs, respectively. The Raman band marked with an asterisk (*) is affected by the contribution of urea. The Raman spectra of the RNAs in droplets and homogeneous solutions are also shown at the top. To obtain spectra of the RNAs in droplets, the spectrum without the RNAs was multiplied by a factor and subtracted from that in the presence of 1000 ng/μL RNA so that the protein band at 640 cm^–1^ completely disappeared. The Raman spectra of polyU in droplets and homogeneous solution were normalized by the intensity at 784 cm^–1^, and those of other RNAs were normalized by the intensity at 1575 cm^–1^.

We measured the Raman spectra of the FUS droplets in the presence of various concentrations of pncRNA31 (Figures 2A (only 0 and 1000 ng/μL) and S3A). When pncRNA31 was added, Raman bands due to the added RNA, such as those at 784 and 1575 cm^–1^, appeared within the droplets alongside the characteristic bands of FUS (the bands indicated with R arise from RNA). No Raman bands due to the RNA were detected outside the droplets (Figure S4), indicating that pncRNA31 was highly concentrated in the FUS droplets. The difference spectrum between 1000 and 0 ng/μL, corresponding to the Raman spectrum of pncRNA31 in the droplets (Figure 2A), exhibited a spectral profile above 1150 cm^–1^ that closely resembled that of pncRNA31 in a homogeneous solution. The Raman bands at 784 and 1092 cm^–1^ exhibited poor agreement in the relative intensity with those of the pncRNA31 solution (Figure 2A). This difference may reflect structural changes in pncRNA31 upon binding to FUS in the droplets.

pncRNA selectively binds to FUS, which may underlie its enrichment within the droplets; however, cRNA and polyU, which lack sequences preferentially interacting with FUS, were also found to be concentrated within FUS droplets (Figures 2B, C and S3B and S3C). The obtained Raman spectra within the droplets closely matched those of the corresponding RNAs in homogeneous solutions (Figure 2B, C). These results suggest that specific RNA sequences are not essential for RNA enrichment within FUS droplets. Similarly, pncRNA50, which is slightly longer than pncRNA31, cRNA, and polyU, was also enriched within the droplets, and its Raman spectrum within the droplets was similar to that observed in a homogeneous solution (Figures 2D and S3D).

We quantified the concentrations of FUS and RNAs inside the droplets and plotted them against the total RNA concentrations (Figure 3A). For the RNA quantification,^45^ the intensity of the 784 cm^–1^ band, normalized to the O–H stretching band of water outside droplets, was used for all the RNAs except pncRNA31, which was quantified using the normalized 1575 cm^–1^ band due to the discrepancy in the relative intensity of the 784 cm^–1^ band between the droplets and homogeneous solution. The RNA concentrations inside the droplets were found to be markedly higher than the total RNA concentration and to increase with increasing added RNA concentration up to 250 ng/μL. This behavior was accompanied by a slight decrease in the FUS concentration, approximately from 8 to 7 mM. The pncRNA31 and cRNA concentrations in the droplets reached 30 μg/μL, and the polyU concentration increased up to about 50 μg/μL. Despite having different sequences and secondary structures, pncRNA31, cRNA, and polyU are found to be concentrated at similar concentrations within the droplets. The maximum concentration of polyU inside the droplets was higher than those of pncRNA31 and cRNA. FUS droplets have been shown to efficiently take up disordered proteins rather than folded proteins,^48^ and the disordered structure of polyU may enable its efficient uptake into FUS droplets. pncRNA50 was also concentrated inside the droplets up to 30 μg/μL (Figure 3A), comparable to pncRNA31 and cRNA; however, its concentration increased more gradually with the total RNA concentration compared to those of pncRNA31, cRNA, and polyU.

**Figure 3:**
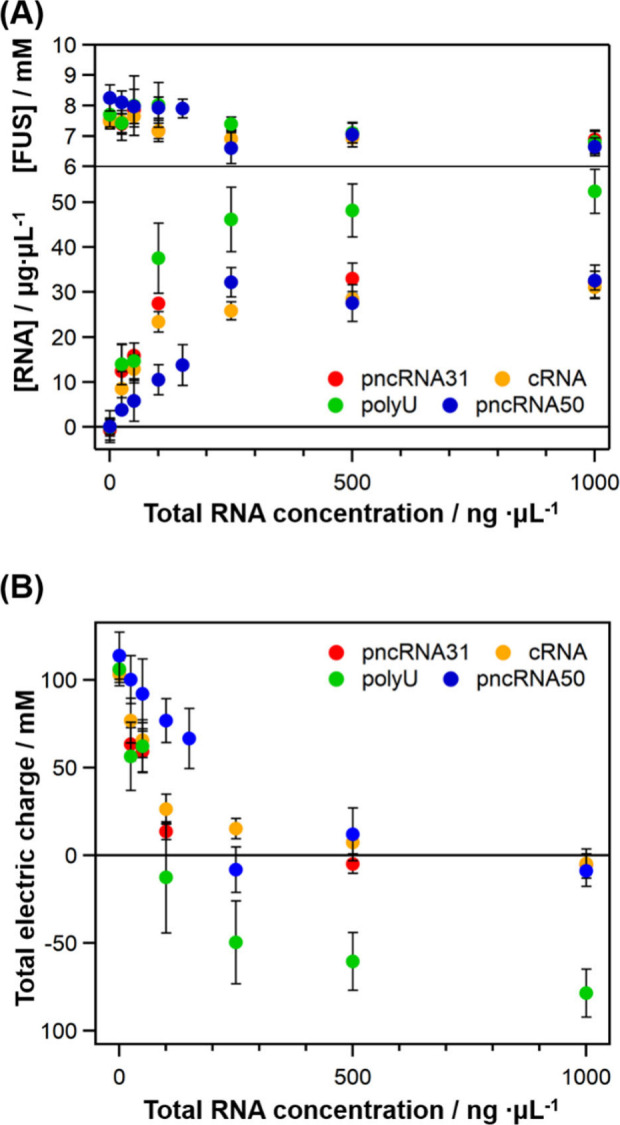
Quantification of (A) the FUS and RNA concentrations and (B) the total electric charge inside the droplets at various concentrations of RNAs added. Error bars are SD. The numbers of measured samples (*n*) are listed in Table S1.

The plots of the RNA concentration inside the droplets exhibit a typical feature of the binding affinity curve, suggesting that the RNAs were concentrated in the liquid droplets through specific interactions with FUS. The RNAs were not concentrated in droplets formed by the low-complexity domain of FUS (FUS LC) (Figure S6), which lacks an RNA-binding domain, suggesting that the RNAs bind to the RNA-binding domain of FUS within the droplets. This result is consistent with a previous mass spectrometry (MS) study, which showed that an RNA (U1-SL34) binds to both the RNA recognition motif (RMM) and the RGG3 region of FUS within droplets.^49^ We predicted the structures of FUS-RNA complexes using AlphaFold3 (Figure S7A-D). All the RNAs were shown to form contacts with the RRM region, and M321 and Y325, which are implicated as cross-linked sites in a previous MS study,^49^ are in the close vicinity of the RNAs (Figure S7E). The zinc finger (ZnF) and the N-terminal side of RGG2 also tend to be close to the RNAs. The geometry of these domains with respect to the RNAs varies, depending on the RNAs investigated.

To investigate whether electrostatic interactions drive RNA uptake into droplets, we calculated the total electric charge inside the droplets at different added RNA concentrations (Figure 3B), assuming the electric charges of +14 for FUS, −31 for pncRNA31, cRNA, and polyU, and −50 for pncRNA50. It is found that the total electric charges of the RNAs, except polyU, approach almost zero at around 250 ng/μL of the RNA concentration, indicating that the RNAs are enriched in FUS droplets to neutralize the positive charge of highly concentrated FUS. Thus, the primary driving force for the incorporation of RNA into FUS droplets is concluded to be the electrostatic interaction. The RRM region is lysine-rich and thus may have a strong contribution to RNA recruitment.

We measured the Raman spectra of FUS droplets in the presence of long-chain nRNA (Figure 4). Even at a total nRNA concentration of 100 ng/μL, just below the threshold causing the droplet dissolution, no Raman signal attributable to nRNA was detected, indicating that nRNA is not concentrated in the droplets. As mentioned above, we demonstrated that the concentration of 50-nt RNA in the droplets increased more gradually with respect to the total RNA concentration compared to 31-nt RNA. These results indicate that the RNA length, rather than the sequence, is the primary factor governing the RNA enrichment in FUS droplets. The effects of the RNAs on FUS droplets and the subsequent aggregate formation can be divided into two categories depending on the RNA length. 50-nt or shorter RNAs are concentrated inside the droplets, stabilizing the droplet state and thereby preventing the aggregation. Long RNAs are not concentrated and instead dissolve the droplets, resulting in the prevention of the aggregation.

**Figure 4:**
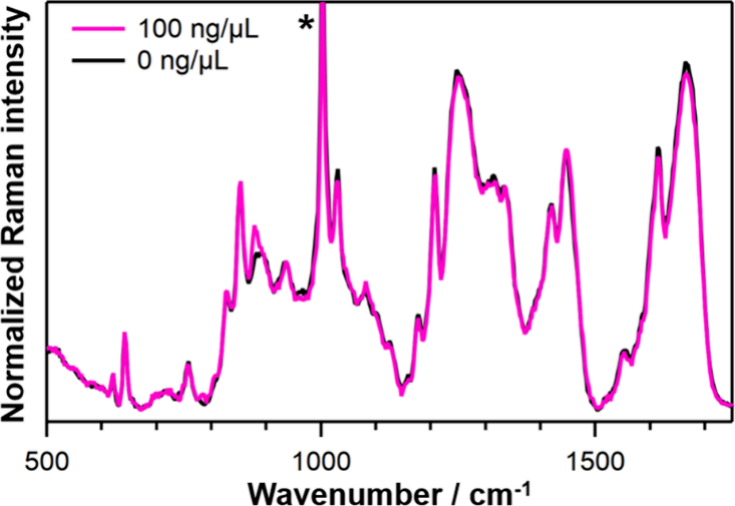
Raman spectra inside FUS droplets with and without 100 ng/μL of nRNA. The spectra were normalized by the Raman intensity of the protein at around 640 cm^–1^. The Raman band marked with the asterisk (*) is affected by the contribution of urea.

### FUS Liquid Droplets in Living Cells Contain High Concentrations of Endogenous RNAs

To investigate whether endogenous RNA is present at high concentrations within FUS droplets in actual living cells, we prepared FUS droplets in HeLa cells and performed Raman imaging of the intracellular droplets in situ (Figure 5). FUS-miRFP670 was expressed in HeLa cells, and the presence of FUS droplets was confirmed by the observation of miRFP670 fluorescence spots within living cells (Figure 5A and B). The droplets were observed predominantly in the nucleus, although a small number were also detected in the cytoplasm.

**Figure 5:**
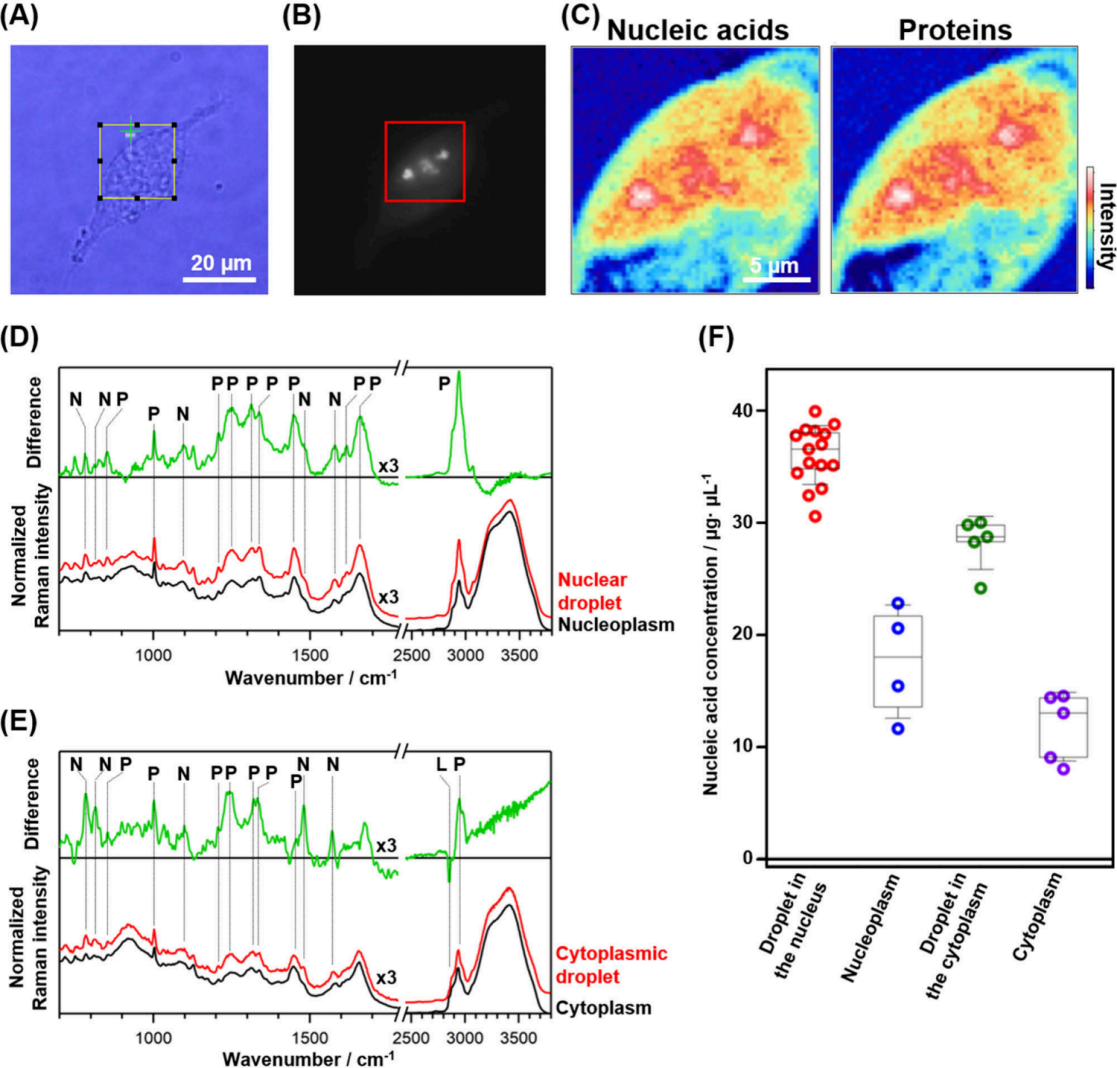
(A) Bright-field, (B) fluorescence, and (C) Raman images of a HeLa cell expressing FUS-miRFP670. Raman images visualize the intensity distribution of the Raman bands of nucleic acids and proteins in the red square shown in (B). (D, E) Raman spectra inside and outside the droplets in the (D) nucleus and (E) cytoplasm. All the spectra were normalized by the Raman intensity of water at around 3400 cm^–1^. The difference spectrum between the inside and outside of the droplets (inside – outside) is also shown in each figure. The Raman bands indicated with N, P, and L arise from RNAs, proteins, and lipids, respectively. (F) The nucleic acid concentrations inside and outside the droplets. Error bars are SD (Droplet in the nucleus: *n* = 15, Nucleoplasm: *n* = 4, Droplet in the cytoplasm: *n* = 5, Cytoplasm: *n* = 5).

Raman images of a cell using the band intensities of nucleic acids at 784 cm^–1^ and proteins at 1002 cm^–1^ showed structures at the same location as the FUS droplets (Figure 5C, Raman images of other HeLa cells are shown in Figure S8). This result indicates that both nucleic acids and proteins are present at higher concentrations within the FUS droplets than in the surrounding intracellular regions. The Raman spectra acquired from FUS droplets in the nucleus and cytoplasm exhibited characteristic bands of proteins, such as the bands at 1002, 1657, and 2940 cm^–1^, as well as bands attributable to nucleic acids, for instance, at 784, 814, 1481, and 1578 cm^–1^ (Figure 5D and E). The Raman band of tyrosine at 1616 cm^–1^ was weaker in intracellular droplets than in droplets formed in a buffer solution, indicating that the droplets in living cells contain endogenous proteins in addition to FUS. The bands observed at 784 and 814 cm^–1^ are characteristic of RNAs, whereas DNAs exhibit only a single band at around 790 cm^–1^ in this wavenumber region. This result indicates that RNA is indeed present at high concentrations in the intracellular droplets. The relative intensities of the two bands at 784 and 814 cm^–1^ in the droplets differ between those in the nucleus and cytoplasm (Figure S9). The low relative intensity of the 814 cm^–1^ band suggests that DNA may also be present inside the droplets in the nucleus.

We compared the Raman spectra of the nucleoplasm and the FUS droplets in the nucleus. The difference spectrum, obtained by subtracting the spectrum of the nucleoplasm from that of the droplets (droplet – nucleoplasm), exhibited positive peaks due to proteins and RNAs (Figures 5D and S9). This result confirms that proteins and RNAs are present at higher concentrations in the droplets than in the surrounding nucleoplasm. In addition, a positive band strongly appeared at 1616 cm^–1^, which was derived from FUS, confirming that the ratio of the FUS concentration to total protein concentration was higher in the droplets than that in the nucleoplasm. The difference spectrum between the cytoplasmic droplets and the cytoplasm (droplet – cytoplasm) also exhibited positive peaks due to proteins and RNAs. In the difference spectrum (droplet – cytoplasm), a negative feature was observed at around 2850 cm^–1^ (Figure 5E). The band at around 2850 cm^–1^ corresponds to the C–H stretching bands of lipids, indicating that lipids are excluded from the cytoplasmic droplets.

We quantified the concentration of nucleic acids inside the droplets (Figure 5F) using the calibration line created by yeast RNA (Figure S5F). The concentrations in the nuclear droplets, nucleoplasm, cytoplasmic droplets, and cytoplasm are 36.0, 18.0, 28.2, and 13.0 μg/μL, respectively. These results mean that nucleic acids are approximately twice as concentrated in the droplets as in the surrounding environment. The nucleic acid concentration in intracellular FUS droplets is similar to that in FUS droplets evaluated in a buffer solution. It is therefore thought that the maximum amount of nucleic acids that enter a FUS droplet is almost constant both inside and outside cells and that nucleic acids enter intracellular FUS droplets to their maximum extent.

To assess the fluidity of intracellular FUS droplets, we carried out FRAP experiments. The droplets exhibited approximately 70% recovery of the near-IR fluorescence within 70 s after photobleaching (Figure S10), indicating that the observed droplets are highly fluid. Fusion events between droplets were also observed (Figure S10), further supporting their liquid-like nature. These findings confirm that intracellular FUS droplets are enriched with RNA and exhibit high fluidity. This observation aligns with our in vitro results, which indicate that the enrichment of RNAs within FUS droplets stabilizes their liquid droplet state.

### Molecular Mechanism of the RNA-Mediated Regulation of LLPS and Aggregation of FUS

We propose a model of how short and long RNAs stabilize and dissolve FUS droplets (Figure 6), respectively, based on the results obtained by Raman microscopy. In the absence of RNAs, homotypic interactions between FUS play a major role in the droplet formation; however, aggregation of FUS is also caused by direct contacts between FUS inside the droplets. Short RNAs are concentrated inside FUS droplets and bind to FUS through electrostatic interactions (Figure 6A). The FUS-RNA heterotypic interactions form networks within the droplets that compete with direct contacts between FUS, thereby stabilizing the droplets and inhibiting FUS aggregation. This result is consistent with a recent study showing that G3BP1, one of the RBPs, suppresses RNA aggregation in droplets condensing a pathogenic RNA repeat by invading the same droplets.^50^ Taken together, the droplets containing both RBPs and RNAs form environments that protect against disease onset, in which their aggregation is mutually inhibited.

**Figure 6:**
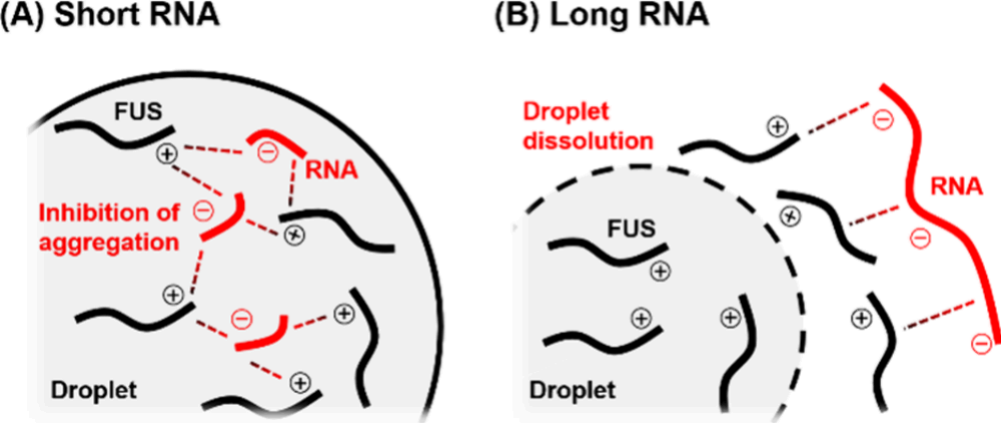
Schematic of the FUS-RNA interactions in droplets.

In contrast, long RNAs were not observed inside FUS droplets, indicating that they are excluded from droplets or homogeneously distributed inside and outside the droplets (Figure 6B). Like short RNAs, long RNAs can bind to FUS through electrostatic interactions. When FUS is bound to long RNAs within droplets or at the droplet interface, it may acquire increased water solubility, leading to droplet dissolution.

## Conclusion

We investigated how RNAs with different sequences and lengths modulate LLPS and aggregation of FUS, a causative protein of ALS. Raman microscopy revealed that short RNAs, regardless of sequence, are enriched within droplets through electrostatic interactions with FUS, whereas longer RNAs are not enriched. Short RNAs suppress FUS aggregation by inhibiting direct contacts between FUS within the droplets, while long RNAs suppress aggregation by dissolving the droplets. So far, limited experimental and theoretical studies have referred to the RNA-length-dependence of the interactions between droplets of neurodegeneration-related proteins and RNAs, and our results highlight the importance of RNA length in the modulation of LLPS and subsequent aggregation. We showed that endogenous RNAs are enriched within FUS droplets in living cells and confirmed that these intracellular droplets exhibit a high fluidity. The endogenous nucleic acid concentration in intracellular FUS droplets was comparable to that in FUS droplets prepared in buffer solutions, suggesting that the RNA-mediated aggregation inhibition observed in vitro also occurs in cells.

Based on our findings and previous studies, it can be concluded that a liquid droplet containing both RNAs and proteins is an environment that protects from diseases by mutually suppressing their aggregation. RNAs invade protein droplets and suppress protein aggregation, while the proteins suppress RNA aggregation by protein-RNA heterotypic interactions. With our previous findings,^45^ the coexistence of RNAs and proteins is likely a general feature of membraneless organelles in cells, and one of its roles may be the mutual inhibition of RNA and protein aggregation.

## Supplementary Material



## Notes

Protocols on sample preparation, Raman microscopy, fluorescence recovery after photobleaching (FRAP), and data analysis, amino acid of FUS and RNA sequences, pncRNA31, cRNA, and pncRNA50, turbidity measurement, Raman spectra of FUS droplets in the presence of various concentrations of RNAs, Raman spectra inside and outside the droplets in the presence and absence of pncRNA31, calibration lines used for quantification of the concentrations of FUS and RNAs, Raman spectra of FUS LC droplets in the presence and absence of polyU, AlphaFold3 predictions of FUS-RNA complexes, magnified intracellular Raman spectra in the 750–850 cm^–1^ region, fluorescence recovery after photobleaching (FRAP) analysis and observation of fusion of FUS droplets in HeLa cells, the number of measured samples (*n*) in Figure 3 (PDF)
