# Supplementary material for: RNA-Mediated Inhibition Mechanism of Liquid–Liquid Phase Separation and Subsequent Aggregation Revealed by Raman Microscopy
Source: JACS Au. 2025 Nov 3;5(11):5749–57. doi: 10.1021/jacsau.5c01234 (PMC12648311; doi:10.1021/jacsau.5c01234)
Supplement: Supplementary file 1 [file au5c01234_si_001.pdf]

Supporting Information

RNA-Mediated Inhibition Mechanism of Liquid-Liquid Phase Separation and Subsequent Aggregation Revealed by Raman Microscopy

Taisei Ogura†, Uchu Matsuura†, Masato Machida, Kaichi Nagai, Shinji Kajimoto, Shinya Tahara*, and Takakazu Nakabayashi*

Graduate School of Pharmaceutical Sciences, Tohoku University, Aoba-Ku, Sendai 980-8578, Japan.

** To whom correspondence should be addressed. E-mail:*

takakazu.nakabayashi.e7@tohoku.ac.jp

shinya.tahara.c6@tohoku.ac.jp

INDEX

Experimental Methods

Sample preparation 3

Plasmid transfection into HeLa cells 4

Raman microscopy 4

Concentration quantification 5

Fluorescence recovery after photobleaching (FRAP) 6

Supplementary Figures

Figure S1. Amino acid sequence of FUS and RNA sequences 7

Figure S2. Turbidity assay in the presence of various RNAs 8

Figure S3. Raman spectra of FUS droplets in the presence of various concentrations of RNAs 9

Figure S4. Raman spectra of the inside and outside of FUS droplets 10

Figure S5. Calibration lines used for quantification of FUS and RNAs 11

Figure S6. Raman spectra of FUS LC droplets in the presence and absence of RNA 12

Figure S7. AlphaFold3 prediction of the structures of FUS-RNA complex 13

Figure S8. Raman images of HeLa cells 14

Figure S9. Magnified intracellular Raman spectra in the 750-850 cm^{-1} region 15

Figure S10. Fluorescence analyses of FUS-miRFP670 in living HeLa cells 16

Supplementary Table

Table S1. The numbers of measured samples (*n*) in Figure 3. 17

Experimental Methods

Sample preparation. Full-length FUS (The amino acid sequence is shown in Figure S1) was purified as described in a previous study with slight modifications.[1] The MBP-FUS_FL_WT plasmid was a gift from Nicolas Fawzi (Addgene plasmid # 98651; http://n2t.net/addgene:98651; RRID:Addgene_98651).[2] *E. coli.* BL21(DE3) strain was transformed with the MBP-FUS_FL_WT plasmid. The cells were lysed by using lysis buffer (50 mM Tris-HCl, 1 M NaCl, 10% glycerol, 1 M Urea, 50 mM Glycine, 2 mM β-mercaptoethanol, 2 mM benzamidine, 1 mM PMSF, protease inhibitor cocktail tablet, 1 w/v% Triton X-100, 10 mM imidazole, and 1 w/v% streptomycin) at pH 7.4. The proteins were purified by Ni-NTA affinity chromatography. The resin was washed by wash buffer (20 mM Tris-HCl, 500 mM NaCl, 10% glycerol, 1 M Urea, 20 mM Imidazole, 2 mM β-mercaptoethanol, 2 mM benzamidine, and 1 mM PMSF) at pH 7.4, and the proteins were eluted by elution buffer, prepared by supplementing 200 mM imidazole to the wash buffer. The obtained MBP-FUS solution was dialyzed against dialysis buffer (20 mM Tris-HCl, 500 mM NaCl, 10% glycerol, 1 M Urea, 2 mM β-mercaptoethanol, 2 mM benzamidine, 1 mM PMSF, and protease inhibitor cocktail tablet) at pH 7.0. The proteins were digested with TEV protease at 25 °C to remove the His and MBP tags, and then purified by Ni-NTA affinity chromatography. The buffer exchange was carried out with a buffer (20 mM Tris-HCl, 500 mM NaCl, and 2 M Urea), and the concentration was adjusted to 1 mM using a centrifugal filter. The full-length FUS solution was stored at -80 °C. LLPS of FUS was induced by mixing the stock solution with dilution buffer (20 mM Tris-HCl, 75 mM NaCl) at pH 7.4, and RNA solutions were then added so that the final FUS concentration becomes 100 μM. The obtained droplet suspension was placed onto a glass-bottom dish (Matsunami) coated with phospholipid polymer, poly [2- methacryloyloxyethyl

phosphorylcholine (MPC) -co-n-butyl methacrylate (BMA)] (PMB80, MPC/BMA=0.8/0.2 mole fraction)[3] provided by Prof. Tomohiro Konno (Tohoku University).[4]

FUS LC (The amino acid sequence is shown in Figure S1) was purified following our previous study.[3] The concentration was adjusted to 1 mM using a centrifugal filter, and the storage temperature was -80 °C. LLPS of FUS LC was induced by mixing with dilution buffer (20 mM Tris-HCl, 150 mM NaCl) at pH 7.4 and RNA solutions. The final FUS LC concentration was 100 μM.

pncRNA31, cRNA, polyU, and pncRNA50 (The sequences are shown in Figure S1) were purchased (FASMAC), and nRNA was extracted from HeLa cells using an RNA purification kit (NORGEN BIOTEK). The dilution buffers were used to prepare 7.6 μg/μL RNA stock solutions.

Plasmid transfection into HeLa cells. To obtain the expression plasmid of FUS labeled with miRFP670, the gene of wild-type FUS C-terminally fused with a $(GGGGS)_4$ linker and miRFP670 was inserted between the *KpnI* and *ApaI* sites of pcDNA3.1(+)-N-HA. HeLa cells were cultured in Dulbecco's modified Eagle's medium supplemented with 10% fetal bovine serum and antibiotics at 37 °C in an atmosphere of 5% CO_2. 10^5 HeLa cells were seeded in 35 mm culture dishes with coverslip bottoms and incubated for 24 h. Transfections of HeLa cells with the plasmid were then performed using Lipofectamine 2000 (Thermo Fisher Scientific). The cells were incubated for 24 h at 37 °C after the transfection. After the incubation, the medium was exchanged with PBS buffer containing 300 mM CsCl to induce LLPS of FUS as described in a previous report.[5]

Raman microscopy. Raman measurements were performed as described previously.[6] Briefly, a confocal Raman system (Nanofinder flex2, Tokyo Instruments), equipped with an inverted microscope (Eclipse Ti2,

Nikon), a spectrograph (MS3504i, SOL) with a grating (600 lines/mm, 600 nm blaze wavelength), and a cooled CCD camera (DU970P-SVF, Andor) was used. A 532-nm laser beam (linewidth < 2 MHz, Sprout Solo, Lighthouse Photonics) was focused onto the sample using a water-immersion objective lens (Plan Apo IR 60XC WI, Nikon) under the microscope. The laser output was 50 mW. Raman signals after passing through a 50 μm pinhole were analyzed by the spectrograph and CCD camera. Typical exposure time for in vitro experiments was 5 s. To acquire Raman images of cells, the sample position was scanned by a piezo stage (NanoControl). 60×60 pixels images were obtained at an interval of 0.3 μm, and an exposure time for each pixel was 0.1 s. The area intensities in the 772-797 cm^{-1} and 975-1024 cm^{-1} regions were used for constructing the Raman images of nucleic acids and proteins, respectively (Figures 5C and S8). To detect liquid droplets containing FUS in cells, the fluorescence of miRFP670 was monitored using an LED light source (excitation wavelength: 621 nm) and an sCMOS camera (CS-53M, Bitran). All the measurements were carried out at a temperature in the range of 23-25 °C.

Background correction was performed separately for the 400-1800 and 2600-3750 cm^{-1} regions of spectra of droplets in vitro. Curves through the points in the wavenumber region absent of Raman bands were obtained by polynomial fitting and were subsequently subtracted from the original spectrum. Each Raman image in cells was processed by singular value decomposition and reconstructed with the first 10 spectral components to reduce noise.

Concentration quantification. The concentrations of cRNA, polyU, pncRNA50, and RNAs in intracellular droplets were quantified as dscribed previously.[6] To quantify the pncRNA31 concentration, we used the Raman intensity at 1575 cm^{-1} after subtraction of a linear function through the points at around 1565 cm^{-1} and 1590 cm^{-1}. FUS concentration in droplets was evaluated using the C-H stretching band at 2940 cm^{-1} as described previously.[3]

Fluorescence recovery after photobleaching (FRAP). FRAP measurements were carried out using a confocal fluorescence microscope (FV1000, Olympus). A 633-nm, 150-μW laser beam was focused onto the droplets using an oil-immersion objective lens (UPlanSApo x100, Olympus) to bleach miRFP670 at a specific region within the FUS droplets for 5 sec. The fluorescence images were continuously recorded at 2-second intervals using a 633-nm, 6-μW laser beam.

(A)

```
  1 MASNDYTQQA TQSYGAYPTQ PGQGYSQQSS QPYGQQSYSG  40
 41 YSQSTDTSGY GQSSYSSYGQ SQNTGYGTQS TPQGYGSTGG  80
 81 YGSSQSSQSS YGQQSSYPGY GQQPAPSSTS GSYGSSSQSS 120
121 SYGQPQSGSY SQQPSYGGQQ QSYGQQQSYN PPQGYGQQNQ 160
161 YNSSSGGGGG GGGGGNYGQD QSSMSSGGGS GGGYGNQDQS 200
201 GGGGSGGYGQ QDRGGRGRGG SGGGGGGGGG GYNRSSGGYE 240
241 PRGRGGGRGG RGGMGGSDRG GFNKFGGPRD QGSRHDSEQD 280
281 NSDNNTIFVQ GLGENVTIES VADYFKQIGI IKTNKKTGQP 320
321 MINLYTDRET GKLKGEATVS FDDPPSAKAA IDWFDGKEFS 360
361 GNPIKVSFAT RRADFNRGGG NGRGGRGRGG PMGRGGYGGG 400
401 GSGGGGRGGF PSGGGGGGGQ QRAGDWKCPN PTCENMNFSW 440
441 RNECNQCKAP KPDGPGGGPG GSHMGGNYGD DRRGGRGGYD 480
481 RGGYRGRGGD RGGFRGGRGG GDRGGFGPGK MDSRGEHRQD 520
521 RRERPY
```

(B)

pncRNA31

GUUAAGAGGGUACGGUGGUUUGAUGACACUG

cRNA

GAUGUGAUGCGUAUGCGUAUGGAUGCGUAGA

pncRNA50

GUUAAGAGGGUACGGUGGUUUGAUGACACUG
AACUAUAUUCAAAAGGAAG

Figure S1. (A) The amino acid sequence of FUS and (B) the sequences of pncRNA31, cRNA, and pncRNA50. In (A), the low-complexity (LC) domain, arginine-glycine-rich (RGG) domain , RNA recognition motifs (RRM), and zinc finger domain are shown in blue, red, orange, ang green, respectively.

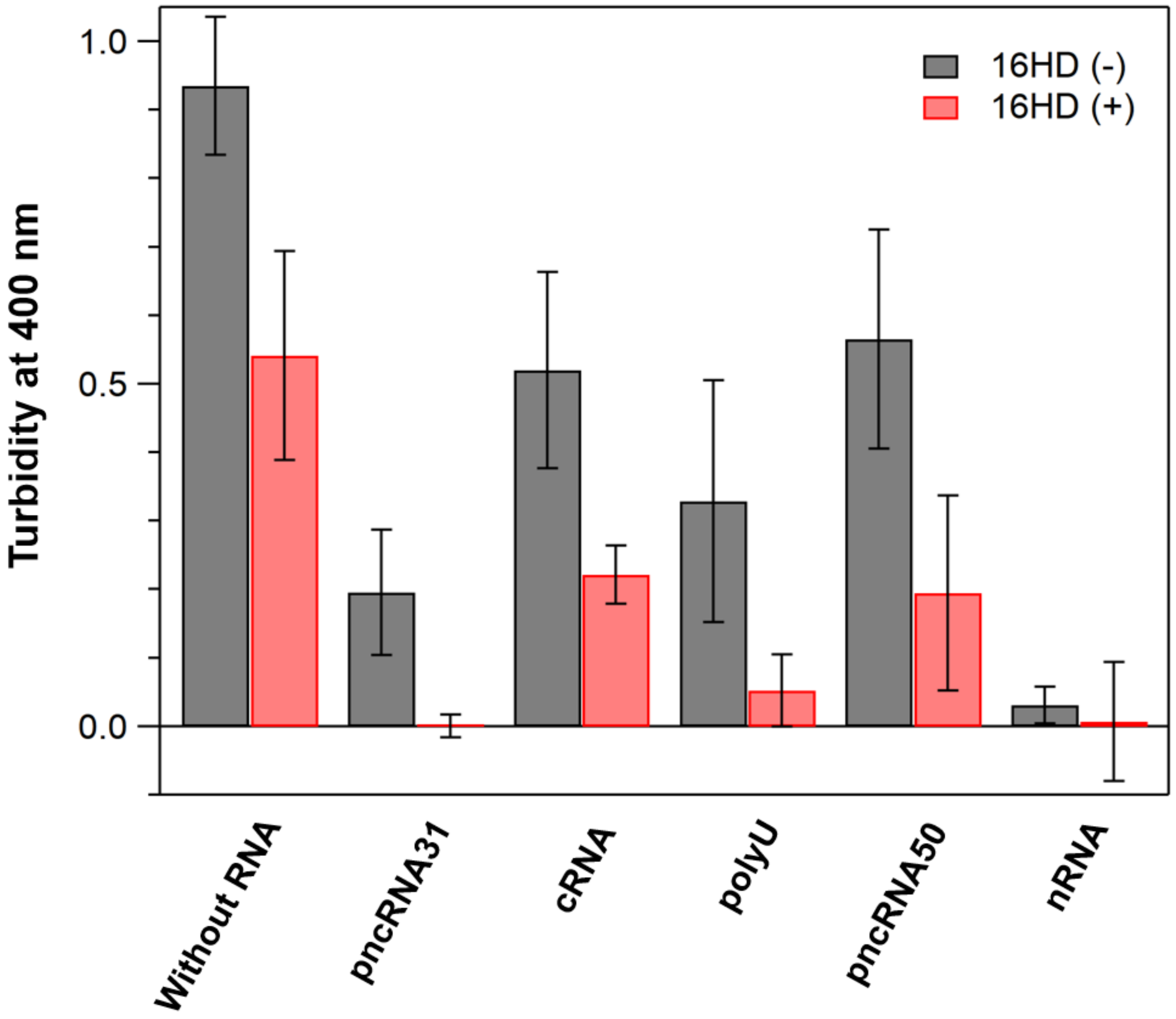

Figure S2. Turbidity at 400 nm after 1 h incubation in the presence of various RNAs with and without 1,6-hexanediol (16HD) treatment. Error bars are SD (n = 3).

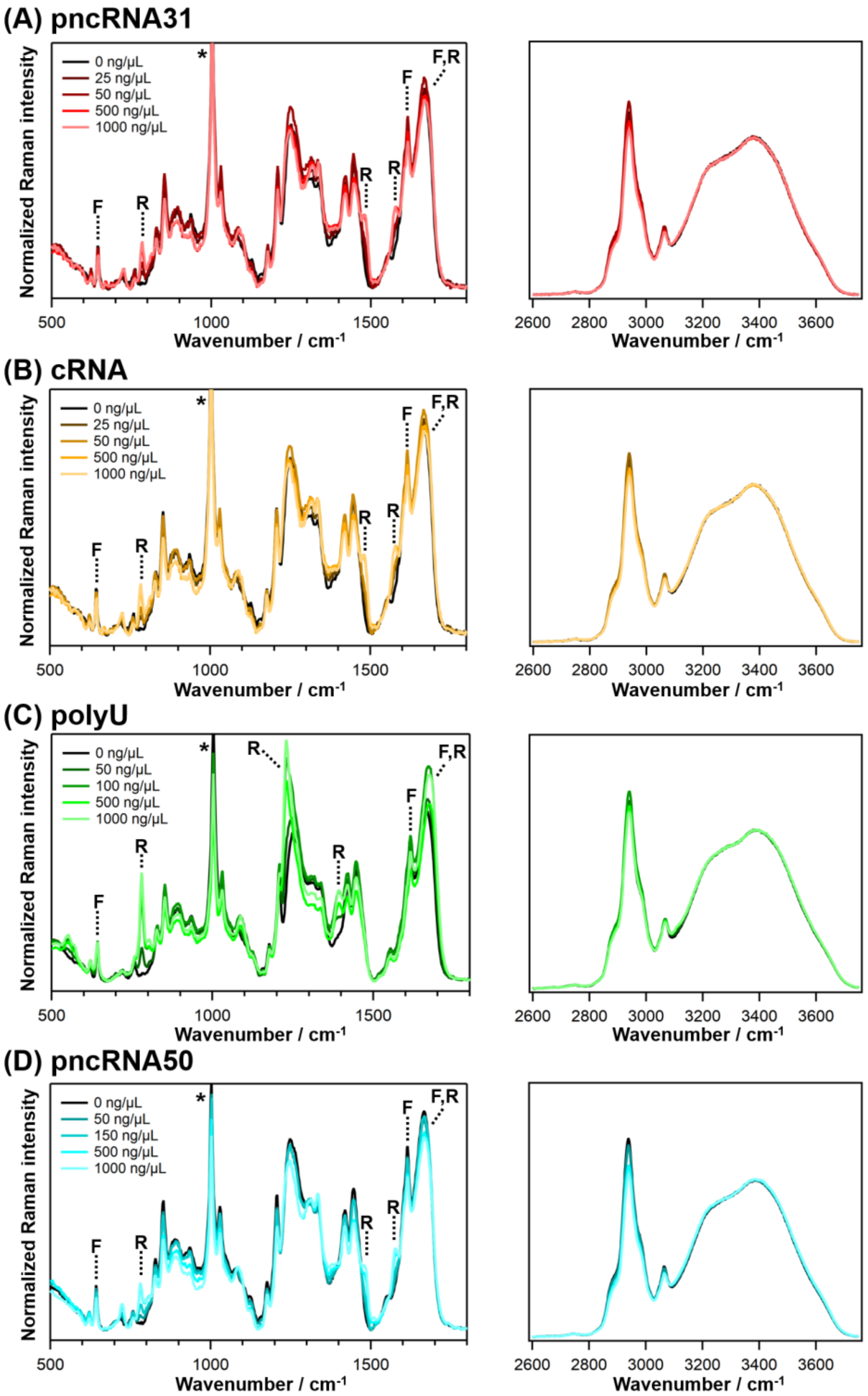

Figure S3. Raman spectra of FUS droplets in the presence of various concentrations of (A) pncRNA31, (B) cRNA, (C) polyU, and (D) pncRNA50. All the spectra were normalized by the Raman intensity of water outside droplets at around 3400 cm^{-1}. The Raman bands indicated with F and R arise from FUS and the RNAs, respectively.

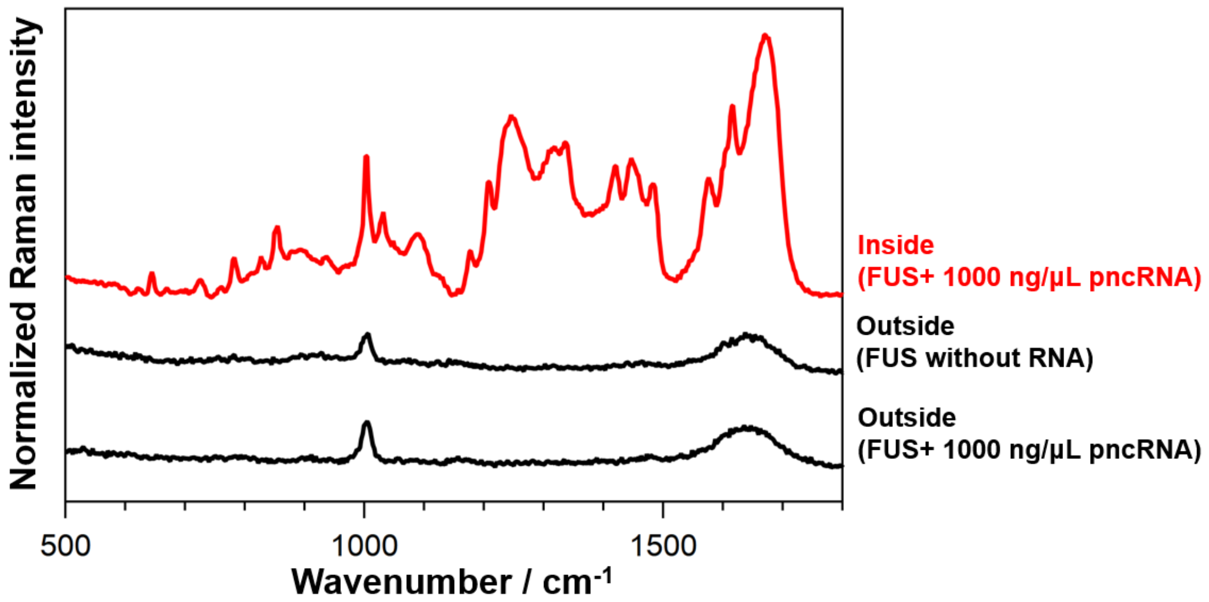

Figure S4. Raman spectra of the inside and outside of FUS droplets in the presence and absence of 1000 ng/μL pncRNA31. All the spectra were normalized by the Raman intensity of water outside droplets at around 3400 cm^{-1}.

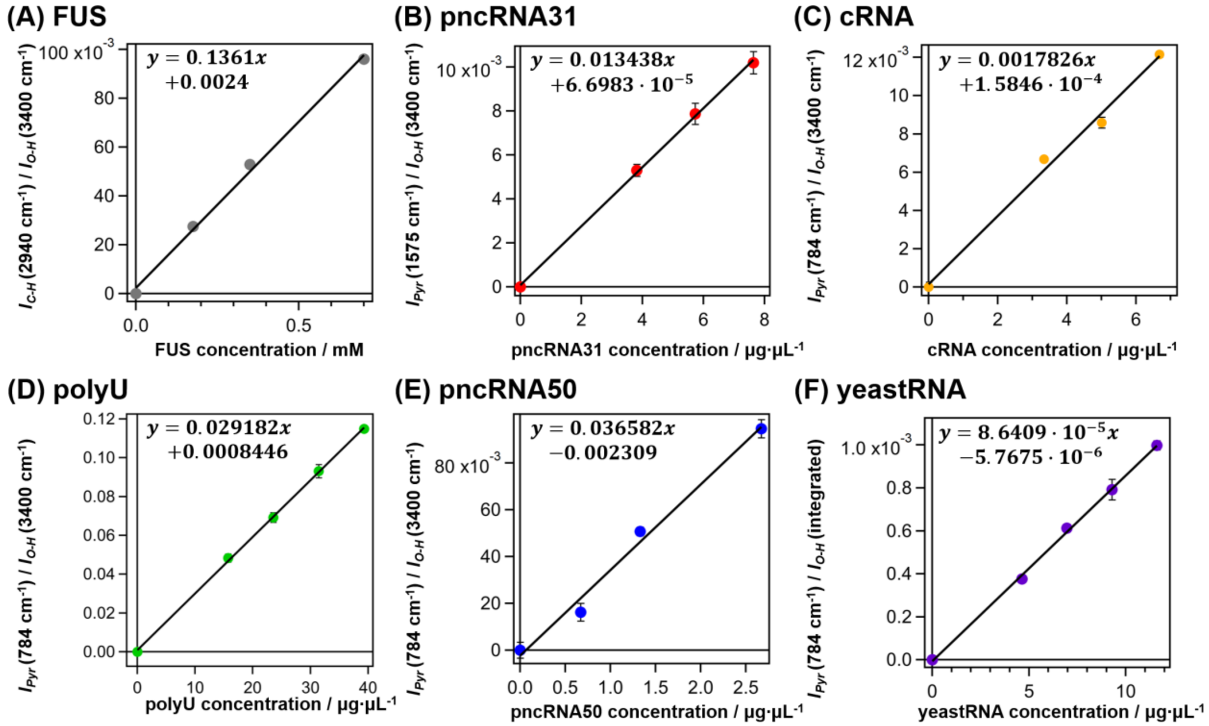

Figure S5. Calibration lines used for the quantification of (A) FUS, (B) pncRNA31, (C) cRNA, (D) polyU, (E) pncRNA50, and (F) intracellular RNA. The lines of FUS, pncRNA31, and the other RNAs were created by plotting the intensity at 2940, 1575, and 784 cm^{-1} normalized by the O-H stretching band intensity of water against the concentrations. We note that the calibration line used for intracellular RNA quantification was constructed by using homogeneous solutions of yeastRNA. Error bars are SD ($n = 3$).

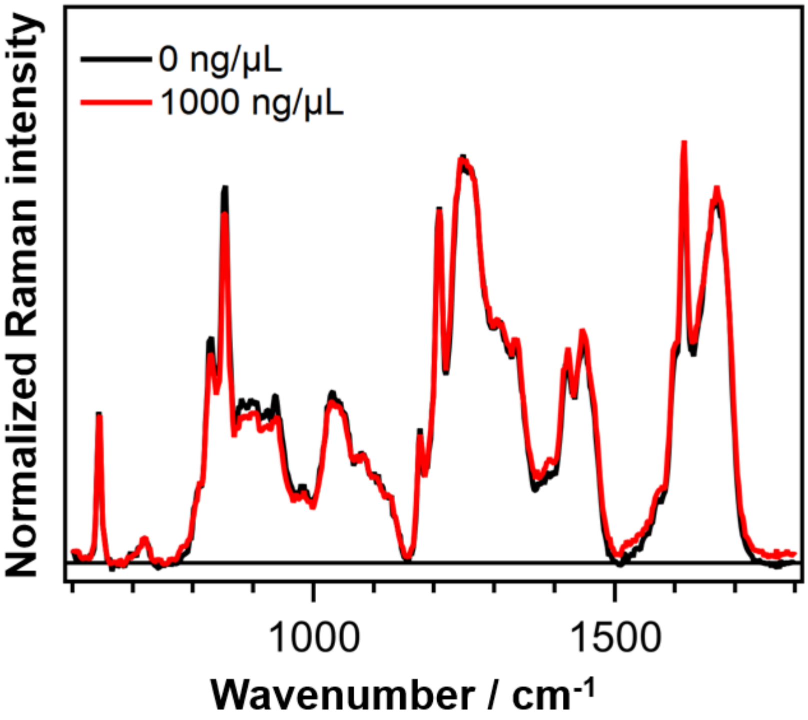

Figure S6. Raman spectra of FUS LC droplets in the presence and absence of 1000 ng/μL polyU in buffer solutions. All the spctra were normalized by the Raman intensity of water outside droplets at around 3400 cm^{-1}.

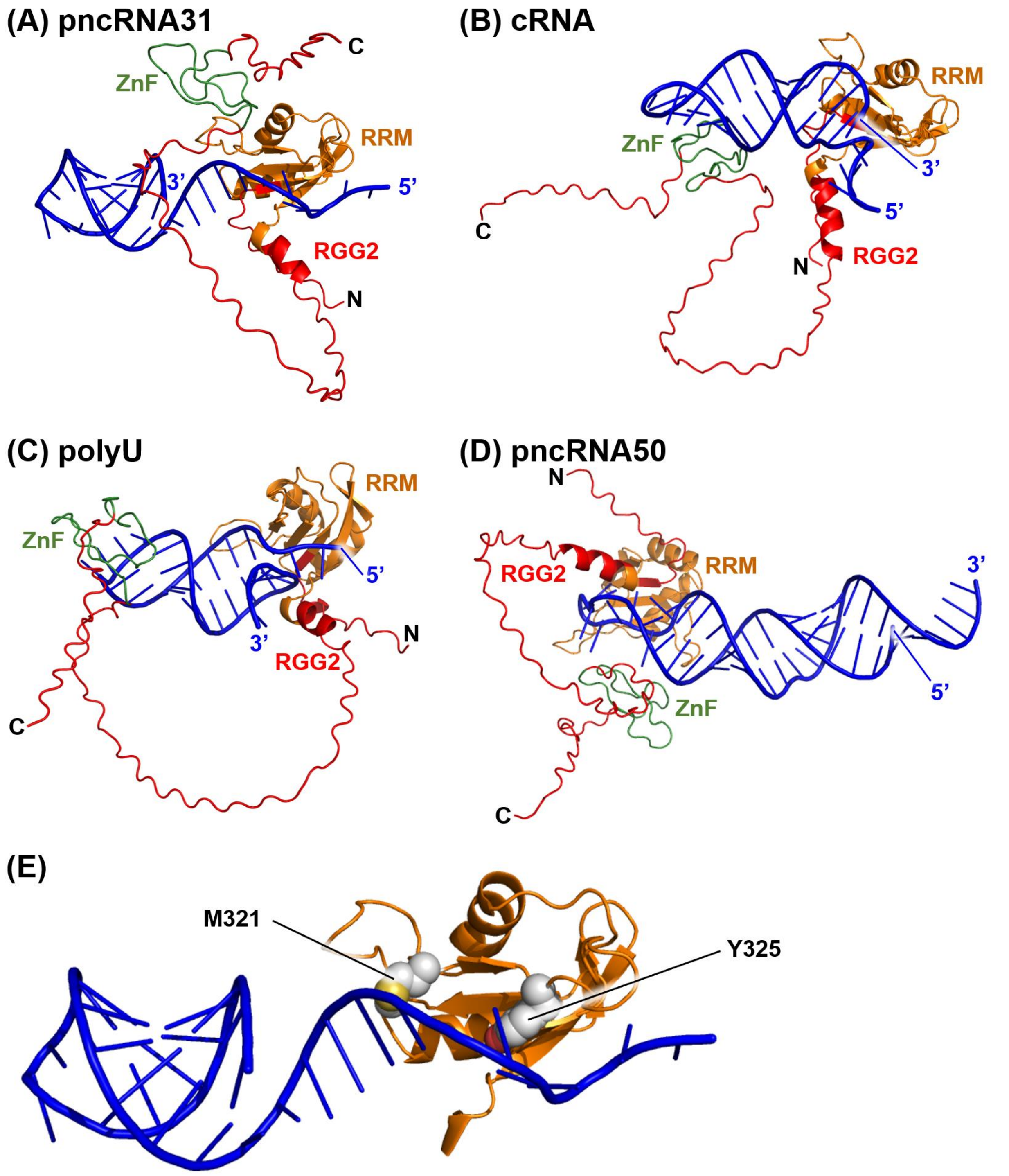

Figure S7. AlphaFold3 predictions of the structures of complexes of FUS (Only residues 271-470 are shown) with (A) pncRNA31, (B) cRNA, (C) polyU, and (D) pncRNA50. The predictions were carried out using full-length FUS (residues 1-526), and only the structures with the highest ranking score are presented. (E) shows the contact region between pncRNA31 and the RRM domain, highlighting M321 and Y325, which were implicated as FUS-RNA cross-linked sites in a previous mass spectrometric study.[7]

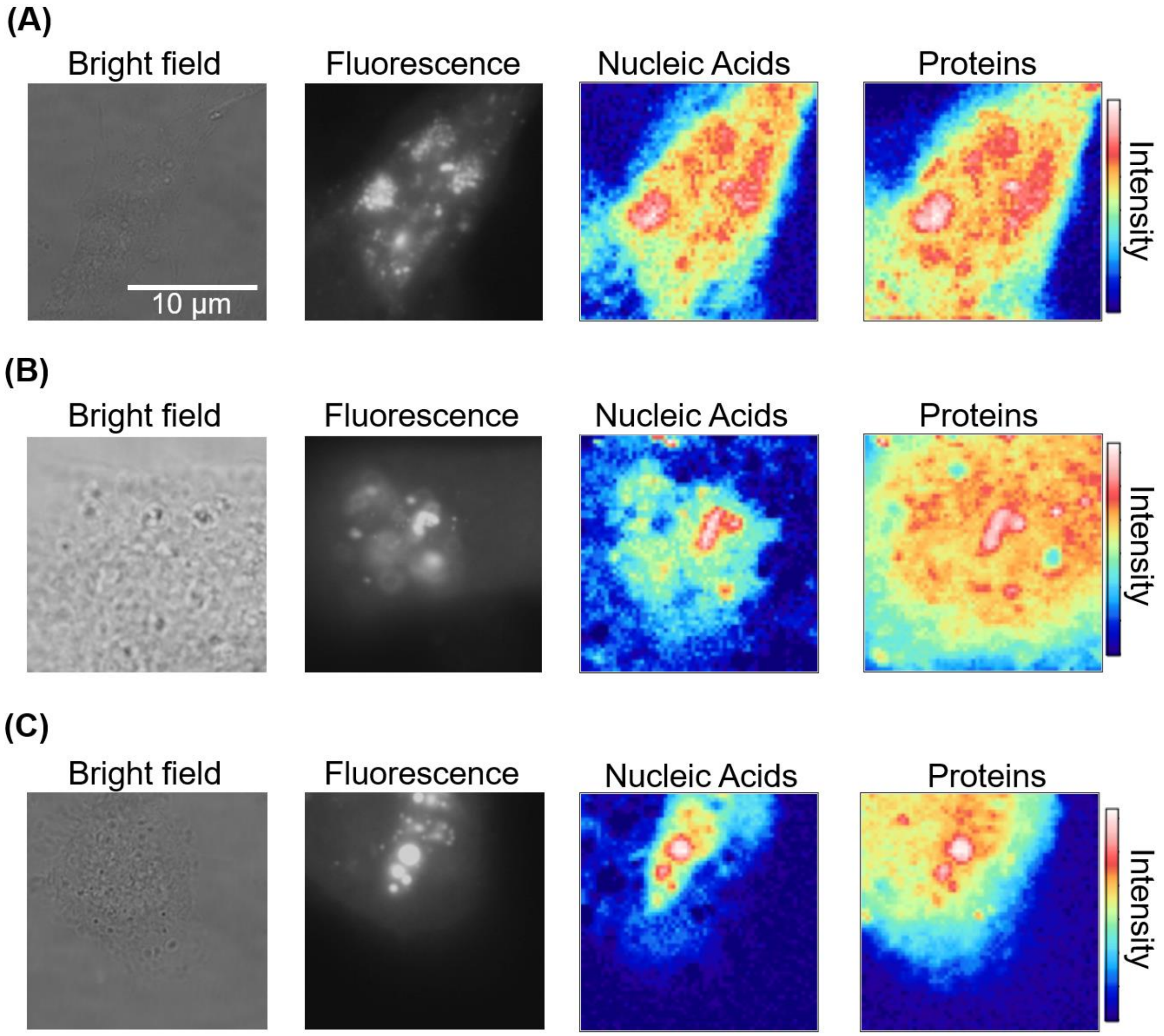

Figure S8. (A-C) Bright-field, fluorescence, and Raman images of three HeLa cells expressing FUS-miRFP670 protein after 1 h incubation with 300 mM CsCl.

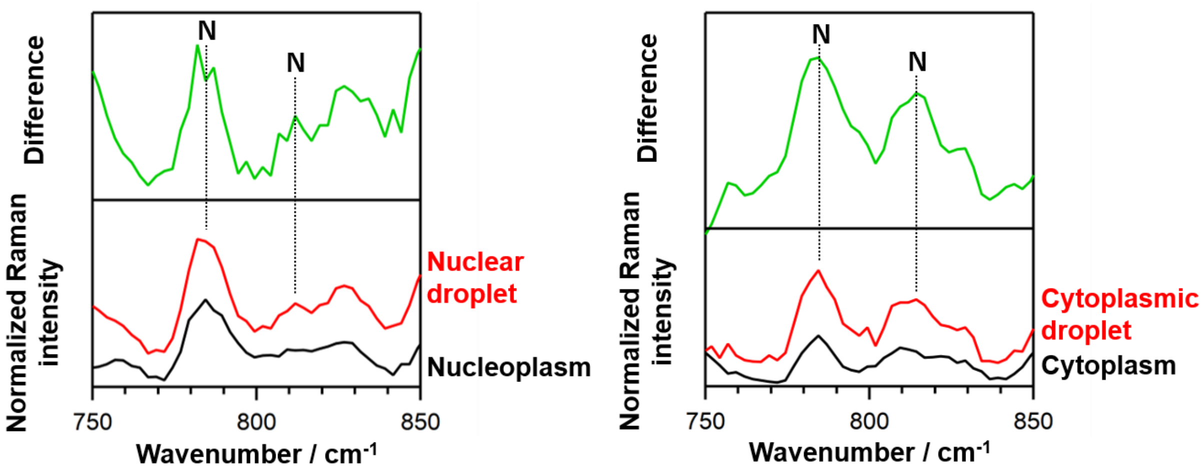

Figure S9. Intracellular Raman spectra in the 750-850 cm^{-1} region. All the spctra were normalized by the Raman intensity of water at around 3400 cm^{-1}. The difference spectra between the inside (red) and outside (black) of the droplets (inside – outside) are also shown by green lines.

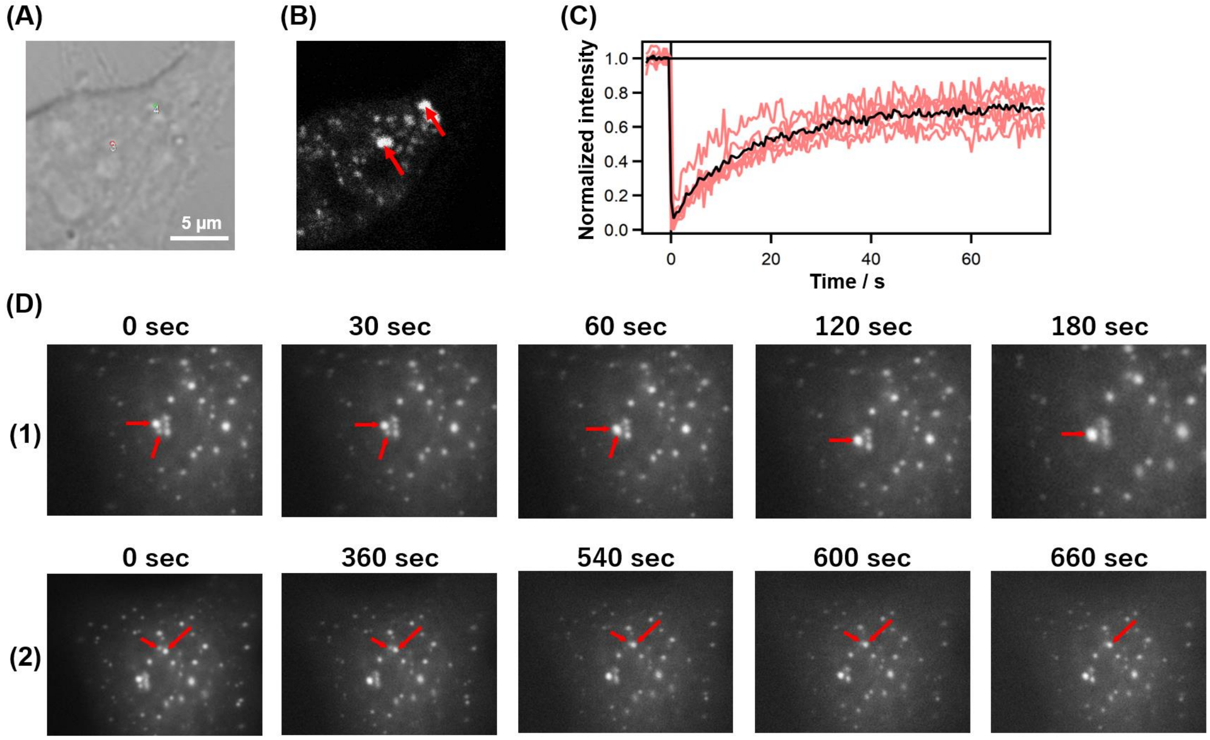

Figure S10. Fluorescence analyses of FUS-miRFP670 in living HeLa cells. (A) Bright-field and (B) near-IR fluorescence images. (C) Fluorescence recovery after photobleaching measurements of two nuclear FUS-miRFP670 droplets indicated with red arrows in (B). Individual and averaged traces are shown in red and black, respectively. (D) Fusion of FUS-miRFP670 droplets in a cell. Two droplets indicated with red arrows undergo fusion. Two independent observations were shown in (1) and (2).

Table S1. The number of measured samples (*n*) in Figure 3.

	Total RNA concentration / ng·μL^{-1}							
	**0**	**25**	**50**	**100**	**150**	**250**	**500**	**1000**
**pncRNA31**	15	15	14	15	-	-	16	21
**cRNA**	18	17	22	17	-	16	16	15
**polyU**	26	20	15	22	-	22	23	19
**pncRNA50**	16	13	16	14	14	17	12	14

REFERENCES

1. Félix, S. S.; Laurents, D. V.; Oroz, J.; Cabrita, E. J., Fused in sarcoma undergoes cold denaturation: Implications for phase separation. *Protein Sci.* **2023,** *32* (1), e4521.

2. Burke, K. A.; Janke, A. M.; Rhine, C. L.; Fawzi, N. L., Residue-by-Residue View of In Vitro FUS Granules that Bind the C-Terminal Domain of RNA Polymerase II. *Mol. Cell* **2015,** *60* (2), 231-241.

3. Yokosawa, K.; Kajimoto, S.; Shibata, D.; Kuroi, K.; Konno, T.; Nakabayashi, T., Concentration Quantification of the Low-Complexity Domain of Fused in Sarcoma inside a Single Droplet and Effects of Solution Parameters. *J. Phys. Chem. Lett.* **2022,** *13* (24), 5692-5697.

4. Ishihara, K.; Mu, M.; Konno, T.; Inoue, Y.; Fukazawa, K., The unique hydration state of poly(2-methacryloyloxyethyl phosphorylcholine). *Journal of Biomaterials Science, Polymer Edition* **2017,** *28* (10-12), 884-899.

5. Zhang, Y.; Xu, N.; Yan, C.; Zhou, X.; Qiao, Q.; Miao, L.; Xu, Z., Live-Cell Imaging to Resolve Salt-Induced Liquid–Liquid Phase Separation of FUS Protein by Dye Self-Labeling. *Chem. Biomed. Imaging* **2024,** *2* (1), 70-80.

6. Shibuya, R.; Kajimoto, S.; Yaginuma, H.; Ariyoshi, T.; Okada, Y.; Nakabayashi, T., Nucleic Acid-Rich Stress Granules Are Not Merely Crowded Condensates: A Quantitative Raman Imaging Study. *Anal. Chem.* **2024,** *96* (43), 17078-17085.

7. de Vries, T.; Novakovic, M.; Ni, Y.; Smok, I.; Inghelram, C.; Bikaki, M.; Sarnowski, C. P.; Han, Y.; Emmanouilidis, L.; Padroni, G.; Leitner, A.; Allain, F. H. T., Specific protein-RNA interactions are mostly preserved in biomolecular condensates. *Sci. Adv.* **2024,** *10* (10), eadm7435.
